# A potent immunomodulatory role of exosomes derived from mesenchymal stromal cells in preventing cGVHD

**DOI:** 10.1186/s13045-018-0680-7

**Published:** 2018-12-07

**Authors:** Peilong Lai, Xiaomei Chen, Liyan Guo, Yulian Wang, Xialin Liu, Yan Liu, Tian Zhou, Tian Huang, Suxia Geng, Chengwei Luo, Xin Huang, Suijing Wu, Wei Ling, Xin Du, Chang He, Jianyu Weng

**Affiliations:** 1 Department of Hematology, Guangdong General Hospital, Guangdong Academy of Medical Sciences, Guangzhou, Guangdong 510080 People’s Republic of China; 2 State Key Laboratory of Ophthalmology, Zhongshan Ophthalmic Center, Sun Yat-Sen University, Guangzhou, 510060 People’s Republic of China; 3 Guangdong Geriatrics Institute, Guangzhou, Guangdong 510080 People’s Republic of China

**Keywords:** Mesenchymal stromal cells (MSCs), Exosome, Chronic graft-versus-host disease (cGVHD), Th17 cells, Treg

## Abstract

**Background:**

Mesenchymal stromal cells (MSCs) are a promising therapy for preventing chronic Graft-Versus-Host Disease (cGVHD) due to their potent immunomodulatory properties. However, the safety concerns regarding the use of MSCs remain unsolved, and conflicting effects are observed due to the heterogeneity of MSCs. Recently, exosomes were shown to mediate the paracrine effects of MSCs, making it a potential candidate for cell-free therapies. The aim of this study is to investigate the efficacy and safety of MSCs-derived exosomes (MSCs-exo) in an established cGVHD mouse model.

**Methods:**

Bone marrow (BM)-derived MSCs were cultured, and the supernatants of these cultures were collected to prepare exosomes using ultracentrifugation. Exosomes from human dermal fibroblasts (Fib-exo) were used as a negative control. The cGVHD model was established, and tail vein injections of MSCs-exo or Fib-exo were administered once per week for 6 weeks. The symptoms and signs of cGVHD were monitored, and histopathological changes were detected by hematoxylin and eosin and Masson staining. The effects of MSCs-exo on Th17, Th1, and Treg were evaluated by flow cytometry, qPCR, and Luminex. In addition, human peripheral blood mononuclear cells (PBMCs) were stimulated and treated with MSCs-exo in vitro. IL-17-expressing Th17 and IL-10-expressing Treg were evaluated by flow cytometry, qPCR, and ELISA.

**Results:**

We found that MSCs-exo effectively prolonged the survival of cGVHD mice and diminished the clinical and pathological scores of cGVHD. Fibrosis in the skin, lung, and liver was significantly ameliorated by MSCs-exo application. In MSCs-exo treated mice, activation of CD4+ T cells and their infiltration into the lung were reduced. Of note, MSCs-exo exhibited potent immunomodulatory effects via the inhibition of IL-17-expressing pathogenic T cells and induction of IL-10-expressing regulatory cells during cGVHD. The expressions of Th17 cell-relevant transcription factors and pro-inflammatory cytokines was markedly reduced after MSCs-exo treatment. In vitro, MSCs-exo blocked Th17 differentiation and improved the Treg phenotype in PBMCs obtained from healthy donors and patients with active cGVHD, further indicating the regulatory effect of MSCs-exo on GVHD effector T cells.

**Conclusions:**

Our data suggested that MSCs-exo could improve the survival and ameliorate the pathologic damage of cGVHD by suppressing Th17 cells and inducing Treg. This finding provides a novel alternative approach for the treatment of cGVHD.

**Electronic supplementary material:**

The online version of this article (10.1186/s13045-018-0680-7) contains supplementary material, which is available to authorized users.

## Background

Chronic graft-versus-host disease (cGVHD) is the primary cause of long-term morbidity and mortality after allogeneic hematopoietic stem cell transplantation (HSCT) [1, 2]. The pathophysiology of cGVHD remains poorly understood, and the therapeutic options for cGVHD have predominantly been limited to steroids and calcineurin inhibitors, which are incompletely effective [3]. Therefore, there is an unmet need to clarify the pathophysiology of cGVHD and develop novel therapeutics for treating this disease.

Chronic GVHD patients present clinical features similar to other autoimmune diseases. Dysregulation of the donor cellular response has been reported to be required in the pathological process of cGVHD [4, 5]. A network of alloreactive T helper cells proliferated, infiltrated, and attacked the targeted organ, leading to the induction of cGVHD. Previous studies have shown the importance of Th1 and Th2 in cGVHD, whereas increasing evidence has indicated that Th17 and Treg cells orchestrate the immunopathological environment in cGVHD [6, 7]. Thus, how to modulate the aberrant T cell response and mitigate the pathologic changes of cGVHD need to be clarified.

Mesenchymal stromal cells (MSCs) have been clinically tested for prophylaxis and treatment of cGVHD due to their potent immunomodulatory properties [1, 8]. Despite their development in clinical therapies, the underlying mechanism for MSC immunomodulatory activity remains tenuous [9]. MSCs inhibit T cell responses as well as modulate the function of B lymphocytes, natural killer cells, and dendritic cells [10]. MSCs regulate the balance of Th17 and Treg and promote transplantation tolerance [4]. In particular, MSCs have been reported to exert their immunosuppressive role mainly through the induction of soluble factors, such as iNOS, IL-10, TGF-b, HGF, PGE2, and IDO, and partially by cell-to-cell interaction [10]. Infused MSCs are short-lived, and the most viable MSCs remain in the lungs [11]. The remote immunomodulatory effect of MSCs in target organs has been regarded to depend on their secretion, which would facilitate the alternative non-cell-based therapies in replace of MSC cell-based therapies.

In addition, increasing evidence has shown that the effects of MSCs are complex and probably fluctuate due to the fascinating biology of MSCs [12]. MSCs are intrinsically heterogeneous and mediate distinct immune modulating responses that are characterized by a pro-inflammatory MSC1 phenotype and an immunosuppressive MSC2 state [13, 14]. Although this view is simplified for the complex process of MSC function, it could explain, in part, the conflicting effects of MSCs in clinical usage. Furthermore, due to the importance of paracrine way in MSC effects, non-cell-based therapies represent an alternative, and the uniformity and standardization of non-cell agents are easier to attain via manufacturing, avoiding the polarization of MSCs in various disease conditions. Moreover, the procedures of non-cell-based therapies are less complicated, and more cell sources are available because immortalized MSC cell lines can be utilized to manufacture. Of note, non-cell-based therapies are safer than cell-based therapies due to their nonviable activity.

Exosomes are a type of nano-level membrane particle that are released from cells and serve as mediators of cell-to-cell communication [15, 16]. MSCs could secrete exosomes to exert their immunomodulatory and regenerative effects [17, 18]. It has been reported that exosomes mediate the paracrine effects of MSCs and promote tissue repair and homeostasis recovery, making them a potential candidate for cell-free therapies. MSC-derived exosomes (MSCs-exo) can recapitulate the therapeutic effects of MSCs in models of myocardial ischemia, acute lung injury/ischemia, and skin wounds [19, 20]. MSCs-exo pass through most physiological barriers due to their small size, allowing effective concentrations to be reached in target tissues [21]. In addition, exosomes can be sterilized by filtration during their preparation for clinical usage. Thus, MSCs-exo exhibit substantial advantages for clinical usage.

To investigate the multifaceted effects of MSCs-exo and interrogate the activity of T cells in the development of systemic cGVHD, we established a murine allogeneic HSCT model and found that MSCs-exo treatment ameliorated the progression of cGVHD. This study indicated that MSC-derived exosomes recapitulated the therapeutic effects of MSCs against cGVHD and possessed the advantages of cell-free therapies.

## Methods

### MSC culture and exosome preparation

Human MSCs were isolated from bone marrow (BM) samples and identified as previously described [1, 4]. Briefly, the bone marrow aspirates (at least 20 mL) were diluted with cultured medium in 1:1 and MSC were isolated with Ficoll-Paque solution (1.077 g/mL; Amersham Biosciences, Uppsala, Sweden) after centrifugation at 800 g for 20 min. The isolated MSC cells were resuspended and cultured at a density of 5000 cells/cm^2^. The medium contains low glucose Dulbecco’s modified Eagle’s medium (L-DMEM; Hyclone, Logan, UT, USA) and 10% fetal bovine serum (FBS; Hyclone). The adherent cells were cultured with medium changes every 3 days. When they were 70–80% confluent, cells were detached by trypsin-EDTA and passaged at a ratio of 1:3 and the third passage MSCs were used for exosome preparations. All these MSCs have been tested for their ability to differentiate into osteoblasts, adipocytes, and chondrocytes. Flow cytometry was performed using a FACSort and analyzed with CellQuest software (Becton Dickinson, San Jose, CA, USA). Only the cells exhibited surface expression of mesenchymal markers (CD73, CD105, and CD166) and cell adhesion molecules (CD29, CD44, and CD90) but negative for hematopoietic markers (CD14, CD19, CD31, CD34, CD45, and HLA-DR) were identified as human MSCs, which fulfill the minimal definition criteria proposed by the International Society for Cellular Therapy. Human dermal fibroblasts purchased from ScienCell were used as control cells. Human dermal fibroblasts were cultured in DMEM/high glucose medium (Invitrogen) that contained 10% FBS, 100 U/ml penicillin, and 100 μg/ml streptomycin at 37 °C.

To manufacture exosomes, cells were cultured with exosome-free FBS for 48 h, which was prepared by a sequential centrifugation procedure as 200×*g* for 10 min, 2000×*g* for 20 min, 10,000×*g* for 30 min, and 110,000×*g* for 7 h at 4 °C, followed by filtration using a 0.22-μm filter [22]. The culture supernatant was collected and performed ultracentrifugation with the same sequential centrifugation procedure as above. The pellet was washed twice with PBS and then filtered through the 0.22-μm filter. The prepared exosomes were stored at − 20 °C until use. The electronic microscopy was utilized for characterization of isolated exosomes. After fixation with 2% paraformaldehyde, the exosomes were negatively stained with phosphotungstic acid for 1 min and examined with a transmission electron microscopy (hitachi H-7650). Markers of exosomes, including CD63, CD9, and CD81, were analyzed by western blot as previously described [23]. The primary antibodies included antibodies against CD63, CD9, and CD81 (Abcam, Cambridge, MA, USA).

### cGVHD mice and treatment

The mouse cGVHD model was established as previously described [24]. Briefly, 10- to 12-week-old BALB/cJ^H-2d^ female mice (Beijing Vital River Laboratory Animal Technology Co., Ltd., China) as recipients received irradiation followed by a tail vein injection of 8 × 10^6^ bone marrow cells and 8 × 10^6^ spleen cells from B10.D2 male mice, the donors purchased from Jackson Laboratories, Bar Harbor, USA. The animal experimental design and procedures were reviewed and approved by the animal experimental ethics committee of Guangdong General Hospital. Recipient mice were monitored every 3 days with respect to the clinical score, body weight loss, and activities beginning at day 14 after bone marrow transplantation (BMT). Mice assigned a clinical score above 0.6 were regarded as established cGVHD. The sry gene on Y chromosome was detected in blood DNA from the female recipient mice on day 20 after BMT. The genotype result showed that all the representative recipient mice presented with sry gene expression, indicating that these mice were indeed transplanted successfully (Additional file 1: Figure S1). On day 22 after BMT, cGVHD mice received a tail vein injection of MSCs-exo or Fib-exo in a 100-μl volume at a dose of 1 μg/μl. The exosome injections were administered once per week for 6 weeks. Blank control mice received equal amounts of a PBS injection. The disease score and skin score were determined as previously described [24], and survival was checked daily for 60 days. The criteria of skin score were briefly determined as follows: healthy appearance = 0, skin lesions with alopecia less than 1 cm^2^ in area = 1, skin lesions with alopecia 1 to 2 cm^2^ in area = 2, and skin lesions with alopecia more than 2 cm^2^ in area = 3. Additionally, animals were assigned 0.3 point each for skin disease (lesions or scaling) on the ears, tail, and paws with minimum score as 0 and maximum score as 3.9. The clinical disease score was based on the clinical manifestation of the skin, body weight, and hunch. When the body weight was loss between 2 and 8%, the mouse had score a point and when more than 8%, the mouse would gain 2 points. For hunch position, the mouse would get a score when hunch quiescent and 2 points when hunch affect action. So the minimum clinical disease score was 0, and the maximum was 7.9.

### Histological analysis and Masson staining

Tissues were fixed with 4% formalin overnight, embedded in paraffin and cut into 6 μm slices. H&E and Masson staining (Masson’s trichrome staining kit, Sigma) were performed separately on consecutive tissue sections, and images were obtained using a microscope (Leica DM4000, Wetzlar, Germany). Quantification of fibrosis was conducted using ImageJ (NIH) as the percentage of blue collagen-stained area relative to the total tissue in one field.

### PBMC culture

PBMCs were isolated from healthy donors and patients with active clinical manifestations of cGVHD, who provided written informed consent in accordance with the Declaration of Helsinki. This experiment was approved by the Ethics Committees of Guangdong General Hospital. Healthy PBMCs were initially stimulated with 2.5 μg/ml PHA (Sigma, USA) and treated with Fib-exo or MSCs-exo (10 μg/ml or 50 μg/ml) for 5 days to detect Treg cells. In addition, other healthy PBMCs were cultured under the inductive condition of Th17 cells (25 μL/well Human T-Activator CD3/CD28 Dynabeads in 24-well plates (Life Technologies, Thermo Fisher Scientific, USA), Il-6100 ng/ml, and TGF-β 20 ng/ml) and treated with individual exosomes to detect the percentage of Th17 cells. Finally, patients’ PBMCs were stimulated with Human T-Activator CD3/CD28 Dynabeads and treated with PBS, Fib-exo, or MSCs-exo (50 μg/ml) for 5 days. The CD4 T subsets were determined by flow cytometry and qPCR.

### Flow cytometry

The cells were isolated from the spleen, lymph nodes (LNs), or lung, and cell surface protein expression was detected and quantified by flow cytometry. A single-cell suspension of lung tissue was prepared to evaluate the infiltration of CD4 T cells. Briefly, the lungs were removed, dissociated, and digested in collagenase D (2 mg/mL) at 37 °C for 30 min. The digested lungs were filtered through 40 mm cell straining to remove the debris. For intracellular cytokine detection, the cells were re-stimulated for 5 h with PMA (20 ng/ml)/ionomycin (1 μM) (Sigma, USA). Golgi-stop was added in the last hour, and intracellular cytokine staining was performed using a BD Biosciences Cytofix/Cytoperm kit as recommended (BD Pharmingen, San Diego, CA, USA). Flow cytometry analysis was performed on a Bectone-Dickinson FACSCalibur (BD Biosciences) using protein-specific monoclonal antibodies as previously described [25]. The data were analyzed using FlowJo software (TreeStar).

### Luminex and ELISA

The relevant cytokines, including IL-17, IL-21, IL-22, IL-2, IL-12, and IL-10, in mouse serum were analyzed using a Luminex MAGPIX system (Luminex Corp., Austin, TX) according to the manufacturer’s instructions [26]. The secretion of IL-17 and IL-10 cytokines from cultured PBMCs obtained from active cGVHD patients was detected by ELISA. The assays were performed using human IL-17A and IL-10 ELISA kits (eBioscience, USA) according to the manufacturer’s instructions. Samples were detected in triplicate relative to standards supplied by the manufacturer and analyzed for significant differences among different groups.

### qPCR

Real-time PCR analysis was performed to detect the mRNA levels of relevant transcription factors as previously described [23]. Briefly, total RNA was extracted with TRIzol (Invitrogen, Carlsbad, CA, USA) and converted into first-strand cDNA using random hexamer primers and the Reverse Transcriptase Superscript II Kit (Invitrogen, Carlsbad, CA, USA). PCR was then performed in a total volume of 20 μL that contained 2 μL of cDNA, 10 μL of 2 × SYBR Premix Ex Taq, 0.8 μL of 50 × ROX Reference Dye (TaKaRa Biotechnology Co., Ltd., Dalian, China), and 10 μmol/L of the primer pairs, which are listed in Additional file 2: Table S1. *Gapdh* was used as a reference gene. The PCR amplification protocol consisted of 95 °C for 30 s and up to 40 cycles of 95 °C for 5 s and 60 °C for 34 s according to the manufacturer’s instructions.

### Exosome labeling assay

Purified exosomes were labeled with a PKH26 red fluorescent labeling kit (Sigma-Aldrich, USA) according to the manufacturer’s instructions. Briefly, the exosomes were incubated with PKH26 dye at a ratio of 5:1 at room temperature for 5 min. After washing with complete culture media (depleted exosomes by ultracentrifugation) and PBS, PKH26-labeled exosomes were isolated by ultracentrifugation at 100,000×*g* for 90 min at 4 °C. A mixture without exosomes was used as the negative control. Then, 10 μg PKH26-labeled exosomes were applied to cultured CD3+ T cells for 12 h. The cells were fixed and stained with Alexa Fluor Phalloidin-488 and DAPI (Invitrogen, USA). Confocal imaging was performed on a confocal microscope (Zeiss LSM800, Germany).

### Statistics

Statistical analysis was performed with SPSS software version 19.0 (IBM, Ehningen, Germany). The data are presented as the mean value ± standard error of the mean (SEM) and were statistically analyzed using a one-factor analysis of variance (ANOVA). Survival curves were plotted as Kaplan-Meier curves and analyzed with log-rank tests. *P* values < 0.05 were considered statistically significant (*P < 0.05, **P < 0.01, ***P < 0.001).

## Results

### MSCs-exo ameliorated cGVHD responses and diminished the clinical and histopathological evidence of cGVHD

**Fig. 1:**
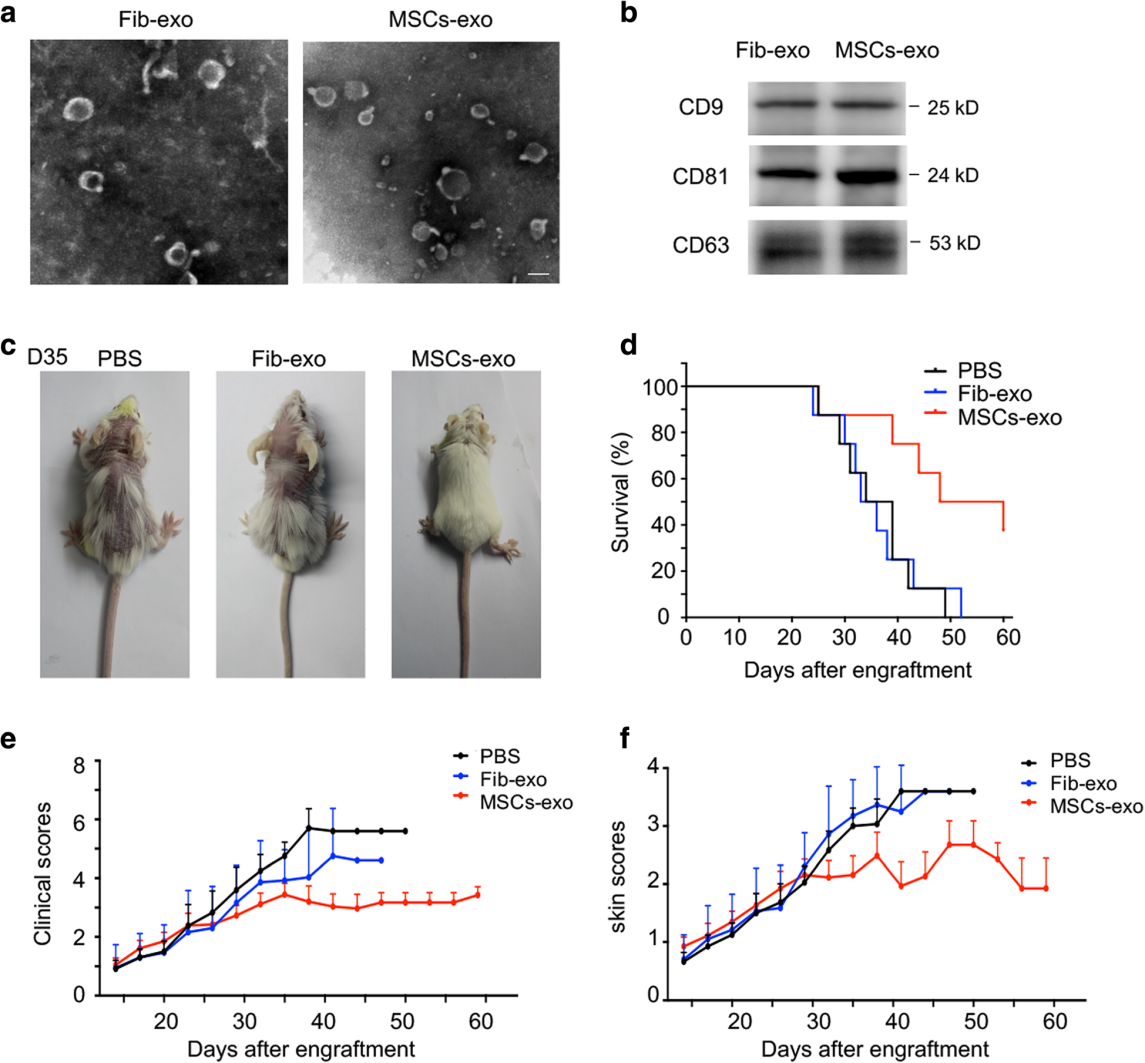
MSCs-exo treatment induced remission of established cGVHD. **a** Representative electron microscopy images of identified exosomes isolated from fibroblasts and MSCs using ultracentrifugation. Scale bar: 100 nm. **b** Both exosomes highly expressed CD9, CD81, and CD63, which were specially enriched in the membrane of exosomes and served as exosome markers. **c** Representative images of cGVHD mice on day 35 after engraftment with typical dermal lesions characterized by hair loss, redness, flaking, scabbing, or hunched posture. MSCs-exo treatment significantly ameliorated these symptoms. **d** Survival curves of mice that underwent allogeneic HSCT treated by PBS, Fib-exo, and MSCs-exo. The survival rates were significantly higher in the MSCs-exo group than in the other groups. **e** The clinical scores (on a scale of 7.9) of cGVHD mice were determined by two researchers in a blind fashion. MSCs-exo-treated mice presented the lowest scores in cGVHD mice. **f** The skin scores (on a scale of 3.9) of cGVHD mice were also determined and the lowest scores were observed in MSCs-exo-treated cGVHD mice. Data were collected from two independent experiments with 6–8 mice per group. Data were expressed as the mean ± SEM

Exosomes derived from MSCs were obtained through sequential ultracentrifugation in this study. A transmission electron image presented the typical rounded shape of an exosome, approximately 100 nm (Fig. 1a). The western blot results showed that Fib-exo and MSCs-exo were positive for CD9, CD81, and CD63, markers of exosomes (Fig. 1b), thereby confirming the presence of exosomes.

To assess the efficacy of MSCs-exo as a therapeutic intervention for cGVHD, we used a typical B10.D2 in the BALB/c mouse of the cGVHD model. Receipt mice were administered tail vein injections of PBS, Fib-exo, or MSCs-exo once per week from day 22 of BMT, when these mice had developed the clinical features of cGVHD. Thirty-five days after BMT, PBS-treated cGVHD mice served as a blank control, which showed clear weight loss and dermal lesions characterized by hair loss, redness, flaking, scabbing, or a hunched posture (Fig. 1c). In contrast to Fib-exo-treated cGVHD mice, which presented similar clinical signs to the PBS-treated mice, mice that received a MSCs-exo injection displayed little clinical features (Fig. 1c). In addition, these MSCs-exo-treated mice had a significant improvement of survival (Fig. 1d) and had the lowest disease scores and skin scores in the cGVHD pathology among all animals (Fig. 1e, f).

**Fig. 2:**
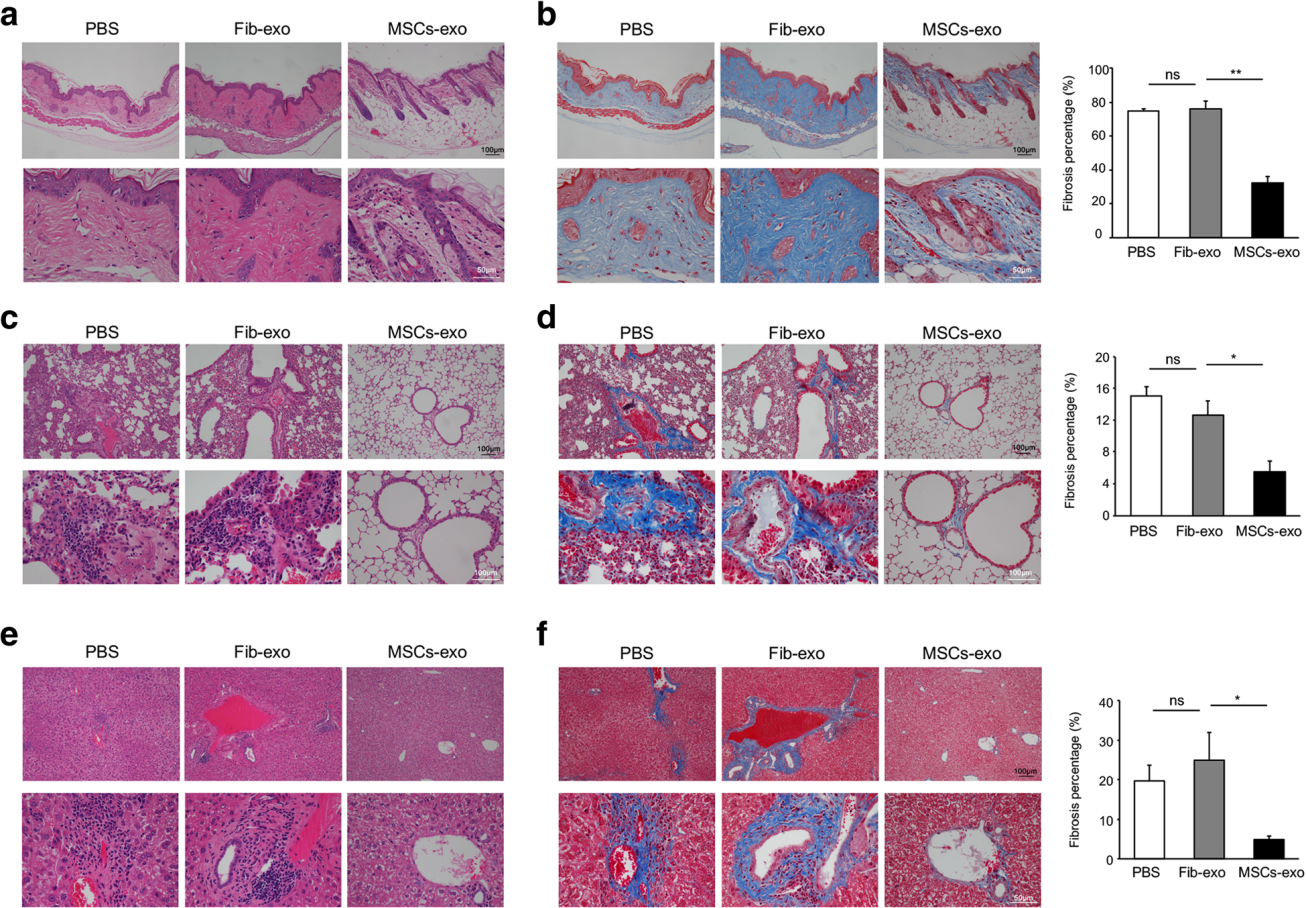
MSCs-exo ameliorated the histopathologic damage of cGVHD mice. **a** H&E-stained skin lesions of cGVHD mice treated with PBS or Fib-exo showed typical dermal fibrosis with a lack of subcutaneous fat and hair follicles, as well as epidermal hyperplasia with increased thickness. By contrast, MSCs-exo-treated mice exhibited reduced levels of dermal fibrosis and hyperplasia. **b** Masson-stained skin section showing obvious fibrosis with collagen deposition in cGVHD mice treated with PBS or Fib-exo, whereas little Masson-positive staining was present in cGVHD mice treated with MSCs-exo. The statistics of Masson-positive fibrosis area percentage also showed least fibrosis in the skin from MSCs-exo-treated cGVHD mice. The fibrosis percentage was determined as the percentage of blue collagen-stained area relative to the total tissue in one upper field. **c** Representative H&E staining images displaying inflammation with an obvious leukocytic infiltration and irregular structure of the lung. However, lung tissue of MSCs-exo-treated mice appeared to be normal with a network of air sacs. **d** Distinct Masson-stained fibrosis and narrow small airways were identified in the lung tissue of control cGVHD mice. By contrast, MSCs-exo-treated mice presented a lack of Masson staining. **e** Representative liver H&E images indicating inflammatory cell recruitment around the hepatic duct in cGVHD mice, and MSCs-exo treatment reduced the infiltration of inflammatory cells. **f** The representative image and statistics showed that fibrosis in the liver was alleviated by MSCs-exo application with reduced Masson staining. Data are expressed as the mean ± SEM. *P < .05 and **P < .01; ns not significant

The observed trends in cGVHD disease were also verified by histopathology. Both cGVHD mice with PBS or Fib-exo application presented typical histopathological features compatible with sclerodermic fibrotic cGVHD (Fig. 2a, b). A thickened reticular dermis and an increase in the extracellular matrix constituents were observed in PBS and Fib-exo-treated mice (Fig. 2a, b). By contrast, MSCs-exo-treated mice exhibited less epidermal fibrosis with a decreased thickness of the dermis and less loss of hair follicles (Fig. 2a, b). The statistics of Masson-positive fibrosis area percentage showed the minimal fibrosis in the MSCs-exo group (Fig. 2b). In addition, these cGVHD mice with PBS or Fib-exo application developed pulmonary cGVHD (Fig. 2c, d). These mice displayed narrower small airways with a significant increase in the peribronchiolar and perivascular collagen deposition (Fig. 2d), indicating the presence of fibroproliferative disease. By contrast, mice treated with MSCs-exo manifested an organized, regular structure of the small airway in the lung, with little Masson-positive fibrosis (Fig. 2c, d). When the liver tissue was analyzed, similar results were observed. cGVHD mice with PBS or Fib-exo treatment presented with severe portal fibrosis and inflammatory cell infiltration, which were significantly suppressed by MSCs-exo treatment (Fig. 2e, f). Overall, these results showed that MSCs-exo had an inhibitory effect on cGVHD.

### MSCs-exo treatment suppressed the activation of CD4 T cells and their infiltration into the lung

**Fig. 3:**
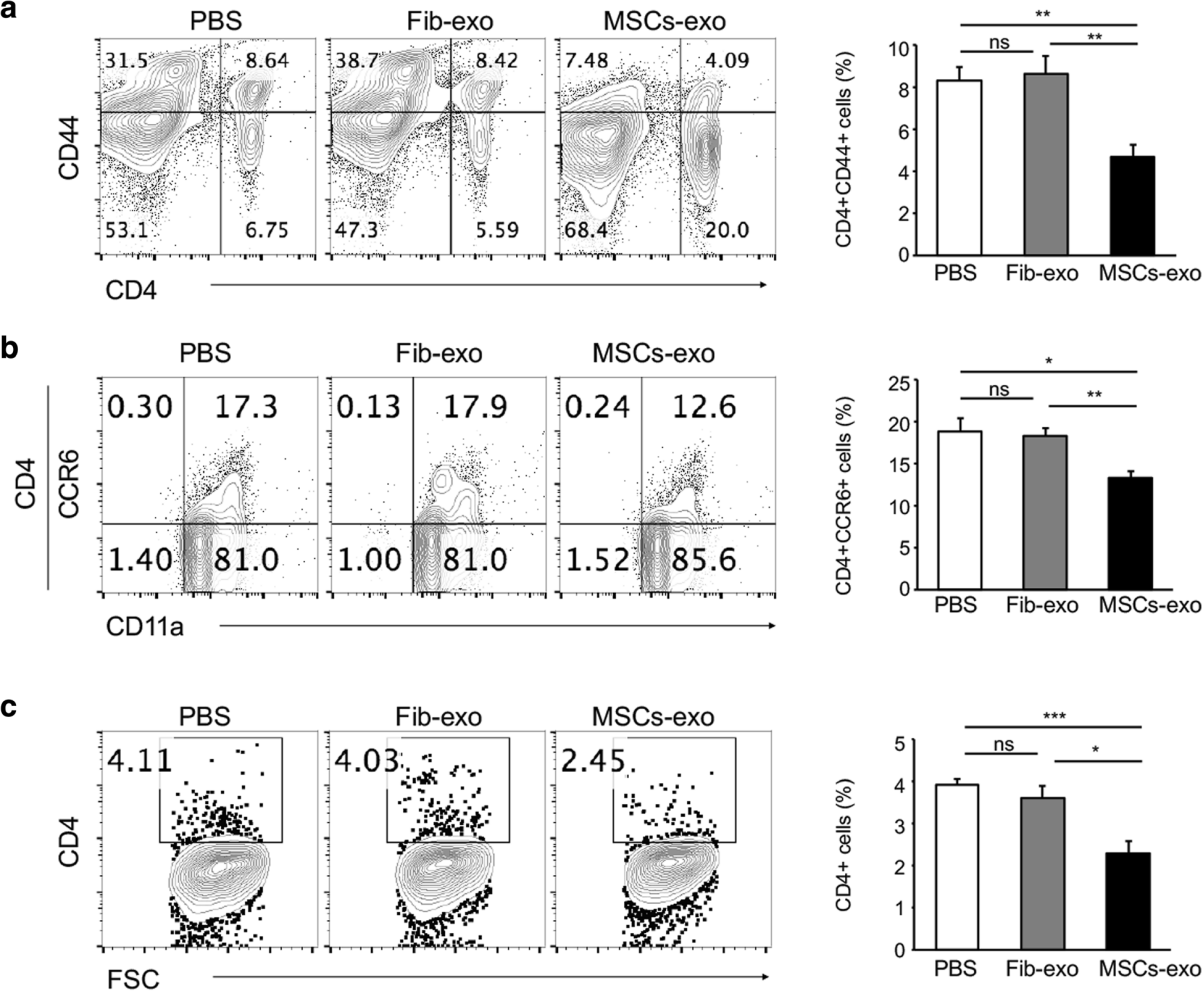
MSCs-exo suppressed the activation and infiltration of lymphocytes into the lung of cGVHD mice. **a** Draining lymph nodes were isolated and analyzed by flow cytometry. MSCs-exo suppressed the expression of CD44, an activation marker of CD4+ T cells, which indicates the immunosuppressive effect of MSCs-exo in cGVHD. The plots in **b** were gated on CD4+ T cells, and the numbers in quadrants indicate the percent of CD4+ T cells expressing CCR6. **c** Lung tissues obtained from mice treated with PBS, Fib-exo, or MSCs-exo were isolated on day 39 after BMT; digested with collagenase; and analyzed for effector CD4+ T cells by flow cytometry. A significant reduction of CD4+ T cells was observed in MSCs-exo-treated mice. Data are representative of three independent experiments for **a**–**c**. Data are expressed as the mean ± SEM. *P < .05, **P < .01, and ***P < .001; ns not significant

It is of note that alloreactive T cells were required for the induction of cGVHD. To understand the therapeutic mechanism of exosomes from MSCs, the activation of effector CD4+ T cells in the lymphocytes of cGVHD mice was first evaluated by CD44 expression using flow cytometry. As shown in Fig. 3a, the percentages of CD4 + CD44+ in lymphocytes from the cGVHD with PBS and Fib-exo treatment were similar, whereas a reduced percentage was observed in MSCs-exo treated mice, which indicates that MSCs-exo inhibited the activation of CD4+ T cells. Furthermore, the expression of CCR6, which facilitates Th17 cell recruitment, was significantly reduced in the MSCs-exo group (Fig. 3b). Considering the involvement of the lung in T cell-targeted organs in cGVHD, single-cell suspensions of lung tissues were prepared, and the infiltration of CD4 T cells was detected. We found a substantial reduction of CD4+ T cells in the lungs of the MSCs-exo treated mice (Fig. 3c), which was consistent with less infiltration of leukocytes, as shown in Fig. 2c. These findings indicate that the MSCs-exo treatment suppressed the migration and infiltration of CD4+ T cells into the target organ during cGVHD.

### MSCs-exo alleviated cGVHD by inhibiting pathogenic T cells and inducing regulatory cells

**Fig. 4:**
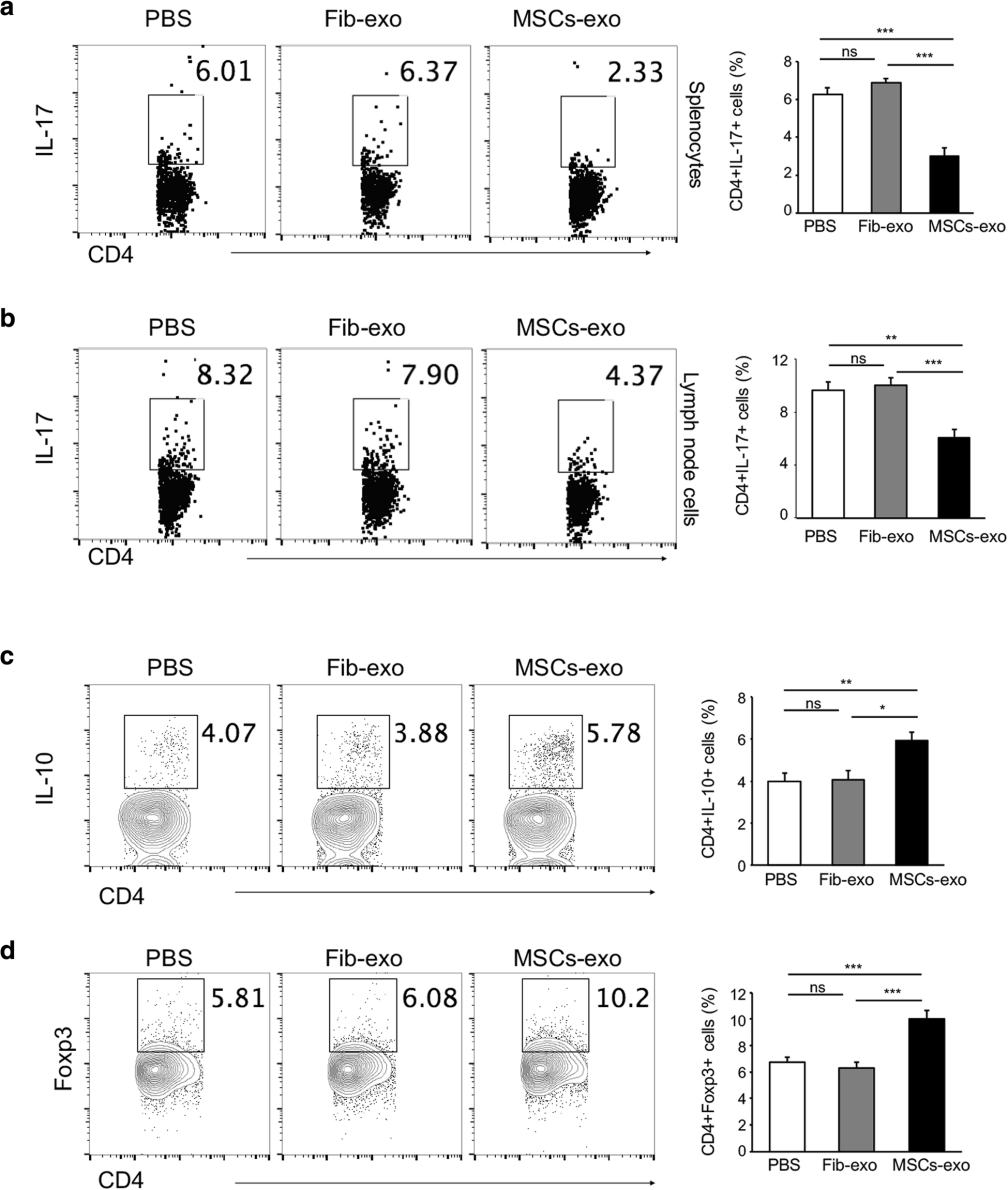
MSCs-exo inhibited IL-17-expressing pathogenic T cells and induced IL-10-expressing regulatory T cells in cGVHD. **a** Splenocytes from individual group mice were isolated and analyzed via an intracellular cytokine staining assay. The numbers in quadrants indicated the percent of CD4+ T cells expressing IL-17. There was a significant reduction of CD4 + IL-17A+ cells in MSCs-exo mice compared with the controls. **b** The lymphocytes from LNs were also analyzed, and the results indicated that MSCs-exo significantly suppressed the development of CD4 + IL-17A+ cells. **c** Treg cells in draining LNs were also analyzed by flow cytometry. As expected, the percentage of IL-10-expressing Treg cells was significantly increased in MSCs-exo-treated mice. **d** The expression of Foxp3, the critical transcription factor of Treg, was also analyzed. There was an obvious induction of Foxp3 expression in the lymphocytes from MSCs-exo-treated mice. Data are expressed as the mean ± SEM. *P < .05, **P < .01, and ***P < .001; ns not significant

Th17 cells are expanded in the spleen and draining lymph nodes of cGVHD mice, and IL-17 has been implicated to be a central mediator of cGVHD pathology. To determine whether the protective effect of exosomes from MSCs was dependent on the Th17-pathway, we collected splenocytes and lymphocytes from LNs and detected Th17 and Treg cells by flow cytometry. As expected, cGVHD is typically accompanied by the presence of IL-17-expressing CD4+ T (Th17) cells (Fig. 4a, b). Strikingly, a significant reduction of Th17 cells was observed in the splenocytes (Fig. 4a) and lymph node cells (Fig. 4b) from MSCs-exo mice compared with the controls. It is interesting to note that the effect was more prominent in splenocytes.

**Fig. 5:**
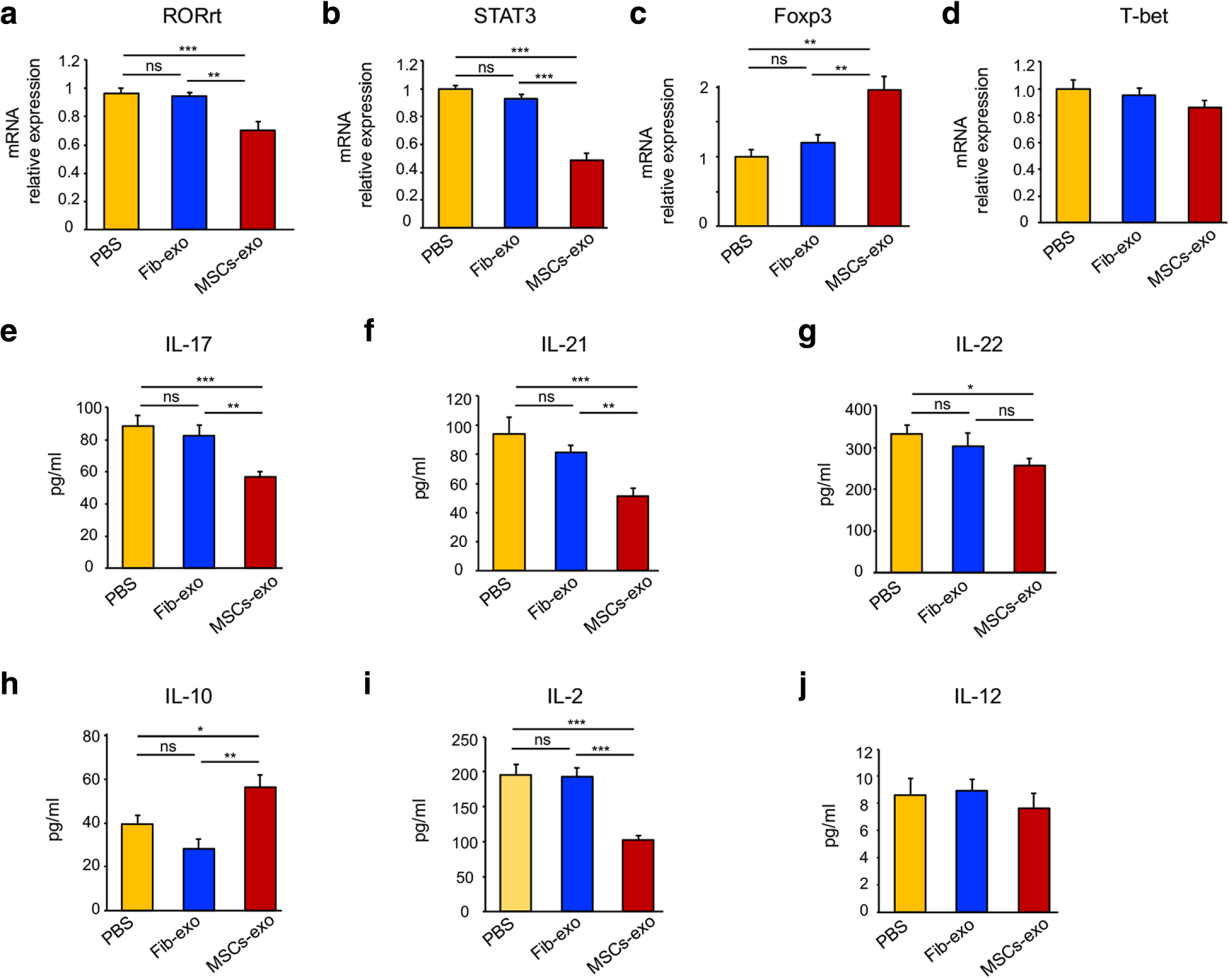
MSCs-exo regulated the expression of transcription factors and inhibited the production of pro-inflammatory cytokines in vivo. **a**–**d** Real-time PCR was utilized to analyze the mRNA expression of transcription factors in PBMC samples from cGVHD mice treated with PBS, Fib-exo, or MSCs-exo. There was no significant difference in gene expression between the PBS and Fib-exo groups, while the MSCs-exo group presented a remarkably reduced expression of RORrt (**a**) and STAT3 (**b**), both of which are involved in the development of Th17 cells, whereas the regulatory gene of Foxp3 (**c**) was increased. No significant difference in T-bet (**d**) gene expression was observed. **e**–**j** A luminex assay was performed to detect the expression level of relevant cytokines. There were significant reductions of IL-17 (**a**), IL-21 (**b**), and IL-2 (**i**) in MSCs-exo-treated mice compared with the controls, while a slight decrease in IL-22 was observed (**g**). By contrast, IL-10 (**h**) expression was increased in the MSCs-exo group. Data are expressed as the mean ± SEM. *P < .05, **P < .01, and ***P < .001; ns not significant

Given the centrality of IL-10 in the suppression of the inflammatory response in transplantation rejection and several autoimmune diseases, we investigated IL-10-producing regulatory T cells (Treg) by flow cytometry. As shown in Fig. 4c, MSCs-exo upregulated the frequency of IL-10-expressing cells. Foxp3, a critical transcriptional factor for Treg differentiation, was also increased in MSCs-exo-treated cGVHD mice (Fig. 4d). In addition, these relevant transcription factors were measured by qPCR. mRNA expression of the detected genes, including RORγt, Stat3, Foxp3, and T-bet, in PBMCs from PBS-treated cGVHD mice was similar to that in the Fib-exo group (Fig. 5a–d), excluding the possible effect of exosomes from fibroblast cells. By contrast, the MSCs-exo treatment induced a significant reduction of RORγt, a transcription factor that is selectively expressed in Th17 cells, and STAT3, which is required for RORγt expression and IL-21 production (Fig. 5a, b). However, T-bet, which is important for Th1 cell development, was almost equivalent between these groups (Fig. 5d), indicating that MSCs-exo exerted no obvious effect on Th1 generation. Taken together, these observations suggested that MSCs-exo inhibited cGVHD by promoting the expansion of Treg cells while inhibiting the pro-inflammatory Th17 cells that mediate cGVHD.

### MSCs-exo treatment reduced pro-inflammatory cytokine production

Th17-relevant cytokine production, also a hallmark of GVHD, was detected in the serum obtained from individual group mice by Luminex. On day 39 after BMT, the expression of IL-17A, IL-21, IL-22, IL-2, IL-10, and IL-12 was similar in serum obtained from the PBS and Fib-exo groups (Fig. 5e–j). By contrast, the MSCs-exo-treated group presented significantly lower levels of IL-17A (Fig. 5e), the typical cytokine produced by Th17 cells; IL-22 (Fig. 5g); and IL-21 (Fig. 5f), which has an essential role in all phases of GVHD pathophysiology to mediate tissue damage. IL-2, a T cell growth factor for amplifying lymphocyte responses, was also reduced in the MSCs-exo-treated group compared with the PBS and Fib-exo groups (Fig. 5i). It is interesting to note that there was no significant difference in IL-12, a cytokine involved in the differentiation of naive T cells into Th1 cells, between the different groups (Fig. 5j). As for the important regulatory cytokine IL-10, we found that MSCs-exo induced an approximately two-fold elevation of IL-10 (Fig. 5h). Thus, MSCs-exo may exert marked immunosuppressive effects on cytokine production.

### MSCs-exo blocked Th17 differentiation and improved the Treg phenotype in PBMCs in vitro

**Fig. 6:**
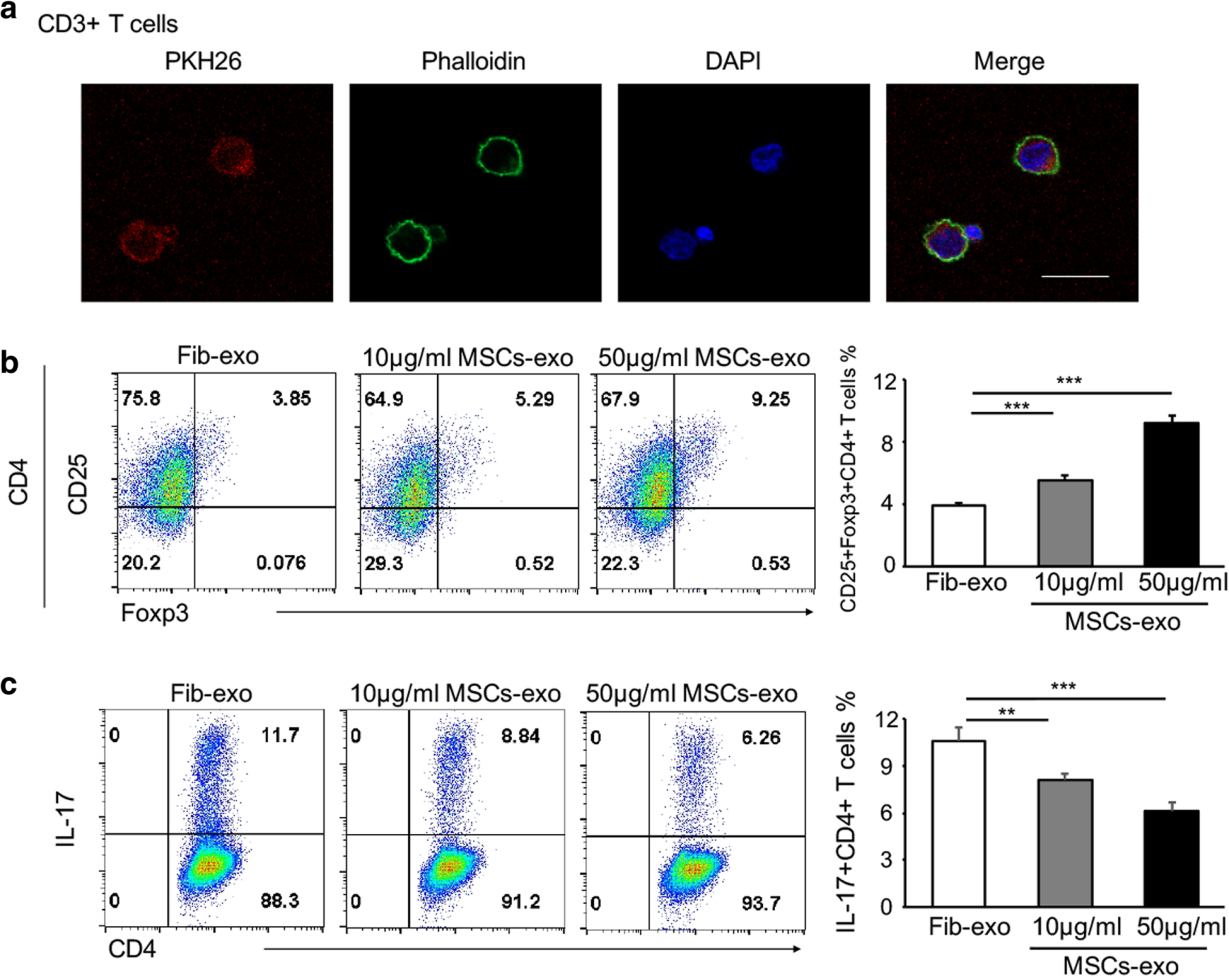
MSCs-exo was uptaken by T cells and regulated cell differentiation. **a** Representative images of exosome uptake by isolated CD3 T cells. **b** PBMCs were isolated from healthy donors and stimulated with PHA and Fib-exo or MSCs-exo (10 μg/ml or 50 μg/ml). MSCs-exo with both doses upregulated the percentage of CD25 + Foxp3 + CD4+ Treg cells. The higher-dose MSCs-exo group with 50 μg/ml presented more Treg cells. **c** When cultured under Th17 culture conditions, MSCs-exo markedly suppressed the differentiation of Th17 cells. Data are expressed as the mean ± SEM. **P < .01 and ***P < .001; ns not significant

**Fig. 7:**
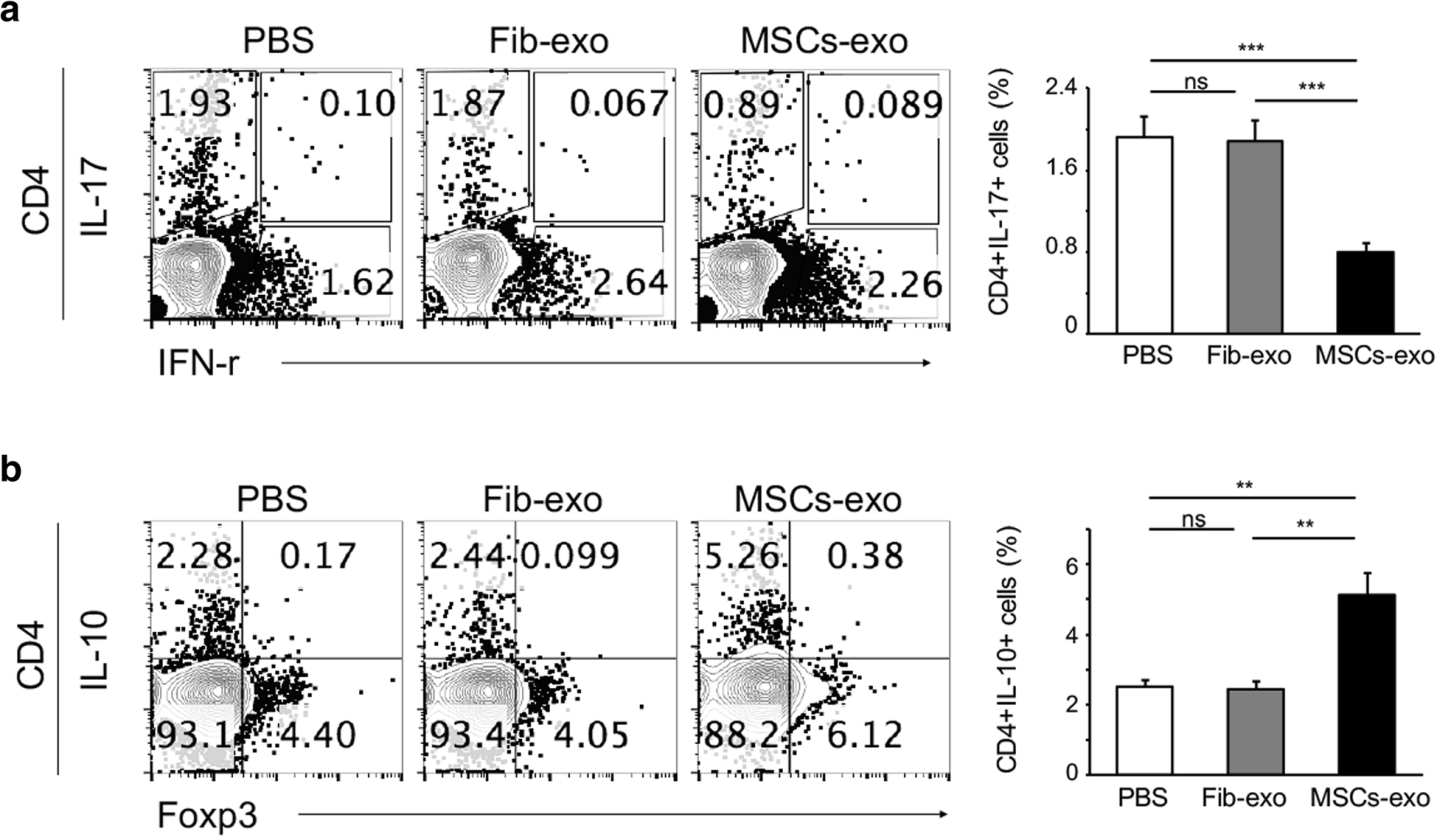
MSCs-exo regulated the differentiation of T cells from patients with active cGVHD. **a**, **b** PBMCs were isolated from patients with active cGVHD, stimulated by T-activator CD3/CD28, and treated with PBS, Fib-exo, or MSCs-exo for 5 days. Consistently, there was a clear reduction of Th17 cells (**a**) in MSCs-exo-treated cells, whereas the percentage of Treg cells was increased (**b**). Data are expressed as the mean ± SEM. **P < .01 and ***P < .001; ns not significant

**Fig. 8:**
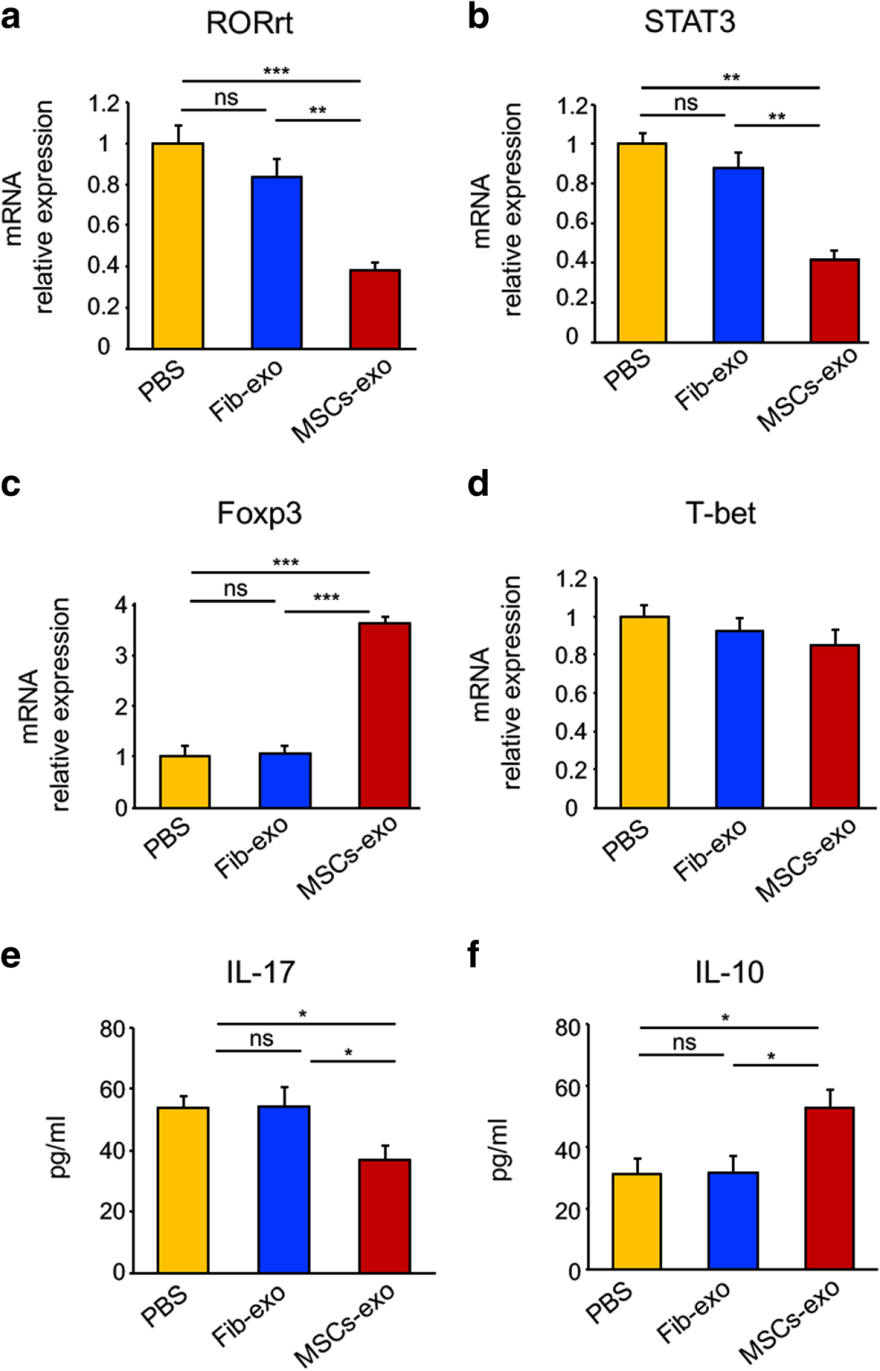
MSCs-exo modulated the gene expression related to Th17 or Treg cells in vitro. **a**–**d** PBMCs were isolated from patients with active cGVHD and cultured udder a stimulative condition. Real-time PCR was utilized to analyze the mRNA expression of transcription factors. As expected, the MSCs-exo treatment suppressed the expression of RORrt (**a**) and STAT3 (**b**), whereas it promoted the expression of Foxp3 (**c**). No significant difference in T-bet (**d**) gene expression was observed. **e**, **f** ELISA was performed to detect the protein expression of IL-17 and IL-10. There was a significant reduction of IL-17 (**a**) and an elevation of IL-10 (**f**) in the MSCs-exo group compared to the PBS or Fib-exo groups. Data are expressed as the mean ± SEM. *P < .05, **P < .01, and ***P < .001; ns not significant

Our data confirm that Th17 cells are critical to the development of cGVHD and that MSCs-exo alleviate cGVHD in murine models, in part, by inhibiting Th17 cells and promoting Treg cells. To confirm that this effect is not restricted to mouse model, we tested the effects of MSCs-exo on human PBMCs in vitro. We initially isolated and cultured CD3+ cells and investigated their uptake of PKH26-labeled exosomes. The actin filaments of CD3+ cells were labeled with phalloidin. As shown in Fig. 6a, PKH26-labeled exosomes were present in the cytoplasm of cultured CD3 cells, which indicates exosome uptake by CD3 cells. Furthermore, MSCs-exo upregulated the percentage of CD25+Foxp3+CD4+ Treg in PBMCs from healthy donors, with a more significant effect in the higher dose MSCs-exo group (Fig. 6b). When PBMCs from healthy donors were cultured under Th17 culture conditions, the differentiation of Th17 cells was markedly suppressed by 50 μg/ml MSCs-exo (Fig. 6c). In addition, PBMCs from patients with active cGVHD were also stimulated with T-activator CD3/CD28 and treated with PBS, Fib-exo, and MSCs-exo. Consistently, IL-17-expressing CD4+ T cells were reduced in the MSCs-exo group (Fig. 7a), whereas the production of IL-10, the anti-inflammatory cytokine that antagonizes pro-inflammatory T cell subsets, such as Th1 and Th17 cells, was significantly upregulated (Fig. 7b). The mRNA expression of relevant transcription factors was detected by qPCR. Similarly, MSCs-exo suppressed the expression of both RORγt and Stat3 and promoted the upregulation of Foxp3 (Fig. 8a–c), further indicating the involvement of the Th17/Treg balance in the mitigated effect of MSCs-exo in cGVHD. Moreover, the typical cytokines IL-17 and IL-10 were evaluated in the supernatants using ELISA. As expected, MSCs-exo inhibited the production of IL-17 while promoted IL-10 generation in PBMCs from human cGVHD (Fig. 8e, f). These data confirmed that MSCs-exo could modulate the development of Th17 and Treg cells in the setting of active cGVHD.

## Discussion

MSC transplantation is undergoing extensive evaluation as a cellular therapy in human clinical trials, and increasing evidence shows that MSCs yield therapeutic effects, largely via the secretion of soluble factors, cytokines, and so on, which has been implicated as the primary mediator of MSC-based therapy [17, 27]. Among the secretion, exosome derived from MSCs is characterized by a small size of 40–150 nm and mediate MSCs [19]. In this study, we found that MSCs-exo attenuated cGVHD and improved the survival of cGVHD mice, extending the usage of MSCs and providing evidence that exosomes derived from MSCs represent a safe and convenient cell-free therapy.

The immunosuppressive effect of MSCs-exo has been evaluated in a mouse model of myocardial ischemia/reperfusion injury, kidney fibrosis, liver injury, and so on [20, 28, 29]. Recently, a study reported that MSC-derived extracellular vesicles could prolong the survival of acute GVHD mice and ameliorate GVHD damage [30, 31]. MSCs-exo alleviated the symptoms of a resistant grade IV aGVHD patient in a preliminary clinical study [32]. Here, we report the dramatic efficacy of MSCs-exo in attenuating cGVHD, a progressive and drug-resistant disease. Of note, the lung complication of cGVHD significantly contributes to the late mortality after HSCT. Lung complications are currently considered diagnostic evidence of cGVHD and re-characterized by frequent non-responsiveness to treatment and irreversibility. In this study, we utilized the B10.D2→BALB/c strain pairing, which uniquely recapitulates key pathologic features of fibrotic human cGVHD in multiple organs, and our data provided evidence that MSCs-exo are a promising therapeutic tool for treating the pulmonary complications of cGVHD.

Interestingly, MSCs-exo exhibited a potent ability to suppress the activation and migration of autoreactive T cells, which participate in the pathogenesis of cGVHD. Activated T cells tend to infiltrate target tissues, resulting in inflammation and tissue damage, which was effectively inhibited by MSCs-exo in this study. CD44 + CD4+ T cells were reduced, and the reduction of IL-17-expressing pathogenic T cells correlated with the decreased expression of CCR6, suggesting that MSCs-exo might mitigate cGVHD by suppressing the trafficking of pathogenic Th17 cells and DP-Th17 cells into target organs during cGVHD. In addition, previous studies have reported that MSCs-exo showed efficacy in treating autoimmune diseases through inhibiting the activation of APCs and T cells [22]. The expansion of Th17 cells is favored by the progressive loss of Treg, leading to cGVHD onset [33, 34]. In humans, the Th17/Treg ratio has been regarded as a specific marker of cGVHD progression [34, 35]. We found that MSCs-exo induced a remarkable reduction of Th17 cells, as well as improved the generation of IL-10-expressing Treg, thereby orchestrating an immunomodulatory condition for immune responses after BMT. Consistently, we found that PBMCs from active cGVHD patients were inclined to Th17 differentiation, which was abrogated by MSCs-exo in vitro. This effect of MSCs-exo was similar to MSCs (data not shown), further indicating that exosome secretion is possibly an important mechanism underlining the suppressive effect of MSCs. However, the underlying mechanism of MSCs-exo in suppressing immunity remains unclear. Exosomes function as a cargo enriched with cytokines, growth factors, signaling lipids, mRNAs, and regulatory miRNAs [15], which then influence the activity of target cells by a variety of mechanisms. It would be very interesting to clarify the specific component of MSCs-exo in treating cGHVD and the molecular mechanism of MSCs-exo modulation of Th17/Treg differentiation in the further study.

Actually, the exosome derived from MSCs have been shown to possess a broad spectrum of immunoregulatory capabilities, such as regulating the function of professional APC and influencing the differentiation and associated cytokine secretion profile of T cell subsets (1,2). In this study, human PBMCs including lymphocytes and APCs were stimulated by anti-CD3/CD28 beads in vitro and MSCs-exo blocked Th17 differentiation and improved Treg phenotype in PBMCs obtained from both healthy donors and patients with active cGVHD, indicating the regulatory effect of MSCs-exo on GVHD effector T cells in the presence of APCs. It would be very interesting to distinguish the effect of MSCs-exo on Th17/Treg in response to various stimulations in the further study.

Our in vivo and in vitro experiments demonstrated that MSCs-exo significantly suppressed the expression of Th17-relevant pro-inflammatory cytokines, including IL-17A, IL-21, IL-22, and IL-2. IL-17A is the signature cytokine generated from Th17 cells, which are the main effector T cells involved in the pathogenesis of cGVHD [6]. IL-21, another cytokine produced by Th17 cells, is also required for the induction of cGVHD, and the blockade of IL-21 has been shown to prevent GVHD [36, 37]. We found that both IL-17A and IL-21 were reduced after MSCs-exo treatment, while little change of IL-22 was observed. These findings indicate the involvement of IL-17A and IL-21 in the immunosuppressive effects of MSCs-exo on cGVHD. Of note, we found that MSCs-exo prominently promoted IL-10 production, which was consistent with previous studies that showed that MSCs-exo induced immune regulatory responses [38, 39].

Exosomes derived from MSCs exhibited potential advantages for clinical usage. First, a non-cell-based therapy using MSCs-exo would be substantially safer than MSC infusion because a non-living agent can avoid the risk of unregulated cell growth and occlusion in the microvasculature [27, 40]. Moreover, there was no death of experimental mice due to vein embolism after tail vein injections in this study. Second, exosomes from MSCs could easily migrate across any physiologic barrier due to their nano-sized level, thereby improving their effect in target tissues [41]. Third, although the available techniques for isolating and purifying exosome remain to be improved, the manufacturing process is less arduous than that of MSCs due to the difficulty of preserving cell viability and function. It would be more amenable to prepare exosomes for clinical usage [42]. Fourth, although exosomes are generated by parent MSCs, they are less likely to trigger an immune response due to the lack of major histocompatibility complex class I/II molecules, rendering them safer to use [43]. As we known, MSCs immunosuppressive ability is not constitutive and these heterogeneous cells could be classified as pro-inflammatory MSC1 and immunosuppressive MSC2 phenotype in response to different TLR-priming stimulation. In addition, there is unavailable standardized protocol to assay the identity of MSC phenotype. In contrast to the heterogeneity of MSCs, exosomes were prepared from MSCs in a controlled and consistent condition such as passage 3 without any TLR-priming stimulation in this study, advancing this therapy into the clinic. Although we did not compare the effects of MSC and MSCs-exo in this study, the substantial efficacy of MSCs-exo and their safety would advance this therapy into the clinic.

Although promising effects of MSCs-exo were observed in the treatment of cGVHD mice, there are several challenges that must be addressed. As exosomes are produced by MSCs, the heterogeneity of which would affect the secretory components, which mediate the immunomodulatory role of exosomes, a standard and scalable cell culture method would be conducive to creating greater consistency in exosomes [42]. In addition, more reliable and efficient techniques for isolating exosomes are warranted for producing cost-effective exosome products [44].

## Conclusion

Our study suggests that exosomes released from MSCs can effectively ameliorate cGVHD in mice by inhibiting the activation and infiltration of CD4 T cells. Furthermore, MSCs-exo exhibits immunomodulatory potency by inducing regulatory T cells and inhibiting Th17 cells. Thus, MSCs-exo provides a new therapeutic paradigm for cell-free MSC-based therapies for cGVHD treatment. Our work supports further investigation into understanding the underlying mechanism so that the intended therapeutic effect can be translated and optimized.

## Additional files

Additional file 1: **Figure S1.** Identification of sry gene in the recipient mice with cGVHD. XY, the positive control of male mice; XX, the negative control of female mice; and G1–G5, five representative mice model of cGVHD. (TIF 320 kb)

Additional file 2: **Table S1.** Primers used for real-time PCR. (DOCX 17 kb)

## Abbreviations

BM: Bone marrow
BMT: Bone marrow transplantation
cGVHD: Chronic graft-versus-host disease
ELISA: Enzyme-linked immunosorbent assay
FBS: Fetal bovine serum
Fib-exo: Exosomes from human dermal fibroblasts
H&E: Hematoxylin and eosin
HSCT: Hematopoietic stem cell transplantation
LNs: Lymph nodes
MSCs: Mesenchymal stromal cells
MSCs-exo: MSCs-derived exosomes
PBMCs: Peripheral blood mononuclear cells
Treg: Regulatory T cells
