# Supplementary figures and images for: A potent immunomodulatory role of exosomes derived from mesenchymal stromal cells in preventing cGVHD
Source: J Hematol Oncol. 2018 Dec 7;11:135. doi: 10.1186/s13045-018-0680-7 (PMC6286548; doi:10.1186/s13045-018-0680-7)

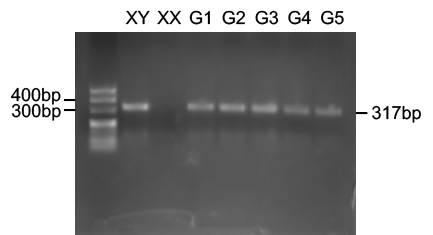

Supplement: Supplementary file 1 — Figure S1. Identification of sry gene in the recipient mice with cGVHD. XY, the positive control of male mice; XX, the negative control of female mice; and G1–G5, five representative mice model of cGVHD. (TIF 320 kb) [file 13045_2018_680_MOESM1_ESM.tif]
