# Supplementary material for: A potent immunomodulatory role of exosomes derived from mesenchymal stromal cells in preventing cGVHD
Source: J Hematol Oncol. 2018 Dec 7;11:135. doi: 10.1186/s13045-018-0680-7 (PMC6286548; doi:10.1186/s13045-018-0680-7)
Supplement: Supplementary file 2 — Table S1. Primers used for real-time PCR. (DOCX 17 kb) [file 13045_2018_680_MOESM2_ESM.docx]

Table S1. Primers used for real-time PCR

| **Gene name** | **Orientation** | **Primer sequence(5’ to 3’)** | **Species** |
| --- | --- | --- | --- |
| *RORγt* | Forward | GACCCACACCTCACAAA TTGA | Mouse |
|  | Reverse | AGTAGGCCACATTACACTGCT |  |
| *Stat3* | Forward | CAATACCATTGACCTGCCGAT | Mouse |
|  | Reverse | GAGCGACTCAAACTGCCCT |  |
| *Foxp3* | Forward | CCCATCCCCAGGAGTCTTG | Mouse |
|  | Reverse | ACCATGACTAGGGGCACTGTA |  |
| *T-bet* | Forward | AACACACACGTCTTTACTTTCCA | Mouse |
|  | Reverse | CGTATCAACAGATGCGTACATGG |  |
| *Gapdh* | Forward | AGGTCGGTGTGAACGGATTTG | Mouse |
|  | Reverse | TGTAGACCATGTAGTTGAGGTCA |  |
| *RORγt* | Forward | CTGCTGAGAAGGACAGGGAG | Human |
|  | Reverse | CACAGAGACAGCACCGAGC |  |
| *Stat3* | Forward | CAGCAGCTTGACACACGGTA | Human |
|  | Reverse | AAACACCAAAGTGGCATGTGA |  |
| *Foxp3* | Forward | GTGGCCCGGATGTGAGAAG | Human |
|  | Reverse | GGAGCCCTTGTCGGATGATG |  |
| *T-bet* | Forward | GATGTTTGTGGACGTGGTCTTG | Human |
|  | Reverse | CTTTCCACACTGCACCCACTT |  |
| *Gapdh* | Forward | ACCCACTCCTCCACCTTTGAC | Human |
|  | Reverse | TGTTGCTGTAGCCAAATTCGTT |  |
| *SRY* | Forward | CGTGGTGAGAGGCACAAGTT | Human |
|  | Reverse | TGTGCAGCTCTACTCCAGTC |  |
